# The Effects of Different Smoking Patterns in Pregnancy on Perinatal Outcomes in the Southampton Women’s Survey

**DOI:** 10.3390/ijerph17217991

**Published:** 2020-10-30

**Authors:** Martin M. O’Donnell, Janis Baird, Cyrus Cooper, Sarah R. Crozier, Keith M. Godfrey, Michael Geary, Hazel M. Inskip, Catherine B. Hayes

**Affiliations:** 1 Mater Misericordiae University Hospital, Eccles Street, D07 R2WY Dublin, Ireland; martinmodonnell@gmail.com; 2 School of Medicine and Medical Science, University College Dublin, Belfield, D04 V1W8 Dublin, Ireland; 3 MRC Lifecourse Epidemiology Unit, Southampton General Hospital, University of Southampton, Southampton SO16 6YD, UK; jb@mrc.soton.ac.uk (J.B.); cc@mrc.soton.ac.uk (C.C.); src@mrc.soton.ac.uk (S.R.C.); kmg@mrc.soton.ac.uk (K.M.G.); hmi@mrc.soton.ac.uk (H.M.I.); 4 NIHR Southampton Biomedical Research Centre, University of Southampton and University Hospital Southampton NHS Foundation Trust, Southampton SO16 6YD, UK; 5 NIHR Applied Research Collaboration Wessex, Southampton Science Park, Southampton SO16 7NP, UK; 6 Department of Obstetrics and Gynaecology, The Rotunda Hospital, D01 P5W9 Dublin, Ireland; mgeary@rotunda.ie; 7 Discipline of Public Health and Primary Care, Trinity College Dublin, D24 DH74 Dublin, Ireland

**Keywords:** smoking, pregnancy, partial quitting, perinatal outcomes, birthweight, gestation, head circumference, crown–heel length, anthropometry

## Abstract

Maternal smoking during pregnancy has established associations with poor perinatal outcomes. Among continuing pregnant smokers, harm-reduction strategies have been suggested, including temporary cessation of smoking during pregnancy, also known as partial quitting. Support for this strategy, however, remains limited. Six hundred and ninety-seven women in the Southampton Women’s Survey who smoked at their last menstrual period were categorised into sustained quitters, partial quitters (quit in either the first or third trimester but not both) or sustained smokers (continued to smoke throughout pregnancy). In regression models, compared with infants born to sustained smokers, infants born to sustained quitters and partial quitters were heavier at birth by β = 0.64 standard deviations (SD) (WHO z-score) (95% CI: 0.47–0.80) and 0.48 SD (WHO z-score) (95% CI: 0.24–0.72) respectively, adjusted for confounders, with similar patterns seen for other anthropometric measures (head circumference and crown–heel length). Sustained quitters had longer gestations by β = 3.5 days (95% CI: 1.8–5.2) compared with sustained smokers, but no difference was seen for partial quitters. While sustained quitting remains the most desired outcome for pregnant smokers, partial quitting should be explored as a strategy to reduce some of the harmful effects of smoking on offspring in those who cannot achieve sustained quitting.

## 1. Introduction

Maternal smoking during pregnancy remains a significant public health challenge. Cigarette smoking during pregnancy has long been related to pregnancy-related complications, such as placenta praevia and placental abruption, and poor perinatal outcomes [1]. Furthermore, maternal smoking during pregnancy has been associated with life-long adverse health outcomes in the offspring, including, but not limited to, diabetes, neurodevelopmental disorders, obesity and respiratory disease [2]. Second-hand smoke exposure during pregnancy has similarly been found to be associated with poorer health outcomes in the perinatal period and later in childhood [3]. These harmful effects are thought to be mediated by the toxic effects of nicotine and carbon monoxide, and perhaps other constituents of cigarettes, which also cross the placental barrier [4].

Worldwide, many women continue to smoke during pregnancy despite being aware of the complications. While the global prevalence of pregnancy smoking is low at around 2%, it exceeds 20% in the United Kingdom (UK) and many European countries, with most women smoking daily and almost 50% smoking more than 10 cigarettes per day [5]. Although socioeconomic gradients for maternal smoking during pregnancy differ by region, in developed countries, rates of smoking during pregnancy tend to be highest among socioeconomically disadvantaged women [6].

Given the known dangers of cigarette smoking during pregnancy on offspring, public health policy is focused on helping women to quit smoking throughout their pregnancy at a minimum and ideally before conception and also post-partum. Approximately half of the pregnant women who smoke spontaneously quit once they become pregnant before their first prenatal appointment [7,8,9]. For the remainder of women, effective smoking cessation interventions during pregnancy consist of non-pharmacological behavioural interventions (such as motivational interviewing and cognitive behavioural techniques) or pharmacological (such as nicotine replacement therapy) [10], both of which reduce smoking rates [11,12].

Women who continue to smoke during pregnancy, the majority of whom are socioeconomically disadvantaged, often decline any of the current interventions for smoking cessation available to them [13]. As such, insight is needed into their experience to allow the development of support specifically designed for them [13]. Women who are socioeconomically disadvantaged may not have the capacity to quit for the duration of pregnancy as they are more likely to lack social support to aid them to quit than more affluent women. Harm reduction, therefore, may be a more attainable goal for this group. Various harm reduction strategies have been proposed, including temporary cessation for a shorter period during pregnancy, also known as partial quitting [14].

Evidence for the effectiveness of partial quitting in improving perinatal outcomes remains limited and needs to be further explored. Birthweight (with the related binary outcomes of low birthweight (LBW) and small for gestational age (SGA)) is associated with adverse childhood growth and psychosocial outcomes [15] and with morbidity outcomes in later life [16,17]. The limited available evidence suggests that partial quitting leads to raw birthweight gains in the order of 150–300 g compared with sustained smoking during pregnancy. Those who quit before the end of the first trimester appear to have birthweights almost comparable to sustained quitting, where gains in the order of 200–400 g have been identified compared with sustained smoking [14,18,19,20,21,22,23,24,25,26,27,28,29]. For LBW/SGA, partial quitting also shows a reduction compared with sustained smoking. In particular, quitting before the end of the first trimester appears to have almost comparable rates to sustained quitting [21,22,26,27,28,29,30,31,32,33].

Shortened gestation (and, in particular, the extreme of preterm birth) adversely impacts neonatal morbidity and mortality, as well as in later life [34]. Large cohort studies have shown a small benefit for partial quitting, albeit not as large as sustained quitting [26,29], although others have found no effect for partial quitting [14,22]. It should be noted that the difference in gestation between sustained smokers and partial quitters in the studies that showed an effect for only 2–5 days, so any absolute difference is likely small. Furthermore, there appears to be a reduction in the rate of preterm birth with partial quitting, especially in quitting before the end of the first trimester in larger cohort studies where in some studies, it is comparable to sustained quitting [21,22,26,27,28,29,30,31,32,35,36].

The World Health Organisation (WHO) recommends the assessment of other anthropometric outcomes, such as head circumference and crown–heel length, which are likely to reflect the intrauterine environment [37]. Maternal smoking during pregnancy is associated with smaller head circumferences and crown–heel lengths [23,24,25,26,27,38,39,40,41]. It is also associated with detrimental foetal measurements of head circumference and other foetal measurements [42]. Quitting by the end of the first trimester appears to have beneficial effects on head circumference and crown–heel length also almost comparable with sustained quitting [26,27]. The benefits of quitting after the first trimester on these variables, however, are conflicting [23,24,25]. The influence of partial quitting on these measures is thus unclear.

Hence, most previously published studies have explored the influence of partial quitting on birthweight and gestation and have seldom included additional anthropometric measures and appear to show the effects of partial quitting on most birth outcomes being somewhere between sustained smoking and sustained quitting.

The study aimed to explore the effects of different smoking patterns during pregnancy (sustained smoking, partial quitting and sustained quitting) on established perinatal outcomes (birthweight and gestational age) further, in a large cohort study adjusting for relevant confounders. A secondary aim was to evaluate the effects of the different smoking patterns on head circumference and crown–heel length, also established and important perinatal outcomes. Our hypotheses were (1) that sustained quitting would have the greatest positive impact on a range of perinatal outcomes, i.e., heavier birthweights, longer gestational age, larger head circumference and longer crown–heel length) compared with sustained smokers who would have the lowest values for these variables, and (2) that for partial quitters, the values for these variables would lie between those of sustained quitters and sustained smokers.

## 2. Materials and Methods

This study consisted of a secondary analysis of the Southampton Women’s Survey (SWS), a longitudinal study of women in the Southampton area of the UK [43]. The SWS received ethical approval from the Southampton and South West Hampshire local research ethics committee (307/97, 153/99w, 005/03/t, 06/Q1702/104, and 10/H0504/30). This study received approval from the SWS Oversight Group for access to the data and secondary analysis.

### 2.1. Study Cohort

The SWS consists of 12,583 women, recruited between 1998 and 2002, aged between 20 and 34 years, of whom 3158 became pregnant and delivered a liveborn singleton infant within the study period. Pre-pregnancy information was gathered from women about their body composition, diet, educational history, lifestyle factors and social circumstances. Data were obtained from expectant mothers during the first trimester of pregnancy (T1) at around 11 weeks’ gestation and in the third trimester (T3) at around 34 weeks’ gestation on their diet, body composition, physical activity and lifestyle factors. Diet was assessed using a food frequency questionnaire [44], the results of which were analysed using principal component analysis to produce a ‘prudent’ (healthy) diet score (as previously reported) [45]. In early pregnancy, women were asked whether they smoked at the time of their last menstrual period. Smoking status was recorded at each visit, and the number of cigarettes smoked was also noted. At birth, anthropometric measures were obtained (with associated World Health Organisation (WHO) z-scores calculated), while gestational age was calculated using a computerised algorithm based on menstrual data and ultrasound assessment in early pregnancy.

### 2.2. Data Definitions

To assess the effect of quitting, all women who were smoking at the start of pregnancy (or last menstrual period) were included. In keeping with the “Russell Standard” [46], quitting was defined as total abstinence since knowledge of the pregnancy or since the previous prenatal visit, and smoking status was based on the women’s self-reported response [47]. Thereafter, smoking categories were derived based on their smoking status at each of the time points, provided full smoking data were available. The categories were defined as follows:

- Sustained quitters: those who quit smoking throughout their pregnancy (i.e., non-smoking at T1 and T3).
- Partial quitters: those who stated they were not smoking at T1 but were smoking at T3 (first trimester quitters) or who stated they were smoking at T1 and not at T3 (third trimester quitters).
- Sustained smokers: those who continued to smoke throughout their pregnancy (i.e., smoking at T1 and T3).

These categories were used in a previous cohort study of partial quitting in an Irish cohort involving two of the authors (C.B.H., M.G.) [14].

### 2.3. Analysis

Descriptive statistics were obtained for the demographic characteristics of the cohort, smoking characteristics and birth outcomes (anthropometric measurements (birthweight, head circumference and crown–heel length) and duration of gestation). Multivariable linear regression analyses with anthropometric measures and gestation as dependent variables were performed, restricted to women who had smoked at the time of their last menstrual period. Sustained smokers were the baseline exposure group. The choice of confounders was guided by Directed Acyclic Graphs (DAGs) [48] (see Supplementary Material—Figures S1–S4), constructed using DAGitty [49]. To reduce biases and adjust appropriately for confounders, the relationships between an exposure, possible confounders and an outcome are represented graphically (as DAGs), and from this appropriate minimal sufficient adjustment, sets can be generated [48,50]. In this study, the DAGs were initially drawn and modified following discussions between the authors until there was agreement. Thereafter, three alternative minimal sufficient adjustment sets were generated for each of the outcomes, all of which shared one minimal sufficient adjustment set (area deprivation, maternal age and maternal education). All outcomes were, therefore, adjusted for this minimal sufficient adjustment set. This minimal adjustment set was also preferred over the others that included variables that potentially were measured less precisely, such as diet and alcohol consumption. Townsend Deprivation Index scores provided the measure of area deprivation. Maternal education was measured in six categories from ‘none’ to ‘degree’ level or above. Data were analysed using SPSS^®^ version 24 (IBM Corp., Armonk, New York, NY, USA).

## 3. Results

### 3.1. Study Population Characteristics

**Figure 1:**
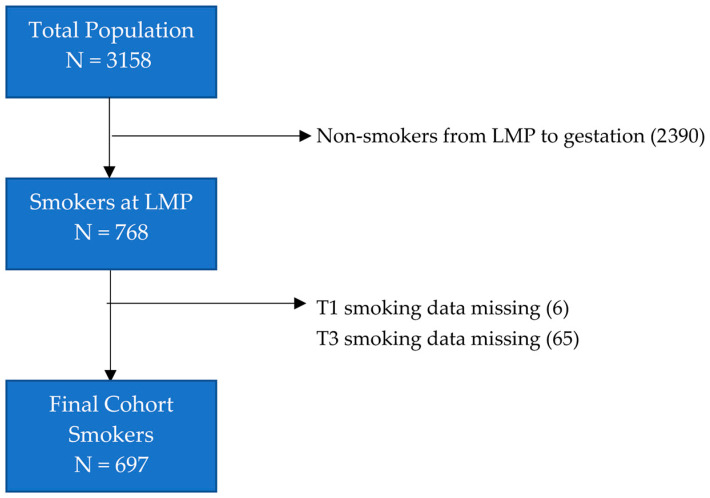
Flow diagram for study participants. Abbreviations: LMP last menstrual period; T1 first trimester; T3 third trimester.

Of the 3158 women who became pregnant during the study period, 768 (24.3%) were smokers at the time of their last menstrual period. Following the removal of individuals for whom there was incomplete data on smoking (see Figure 1), 697 smokers (90.7%) were analysed.

The demographic characteristics of non-smokers and smokers are shown in Table 1. Smokers were categorised according to whether they were sustained smokers, partial quitters or sustained quitters. Smokers tended to be younger, more parous and more socioeconomically disadvantaged compared with non-smokers (as evidenced by higher Townsend Deprivation Index scores, higher rates of receipt of government benefits and lower educational attainment). Sustained smokers appeared to be most disadvantaged, with partial quitters less so and sustained quitters the least disadvantaged. Notably, there was a higher proportion of participants with gestational diabetes mellitus in the non-smokers group (1.5%) compared with the smokers (0.3%).

With regard to maternal smoking characteristics (see Table 2), all groups started smoking at similar ages, and women reduced the number of daily cigarettes smoked during pregnancy regardless of their smoking categorisation. Sustained smokers appeared to smoke more before and during pregnancy compared with those who quit at some point in their pregnancy.

### 3.2. Perinatal Outcomes

The perinatal outcomes are summarised in Table 3 for the different smoking groups. Sustained quitters delivered infants with higher birthweights than partial quitters whose infant birthweights were higher than for sustained smokers who had the lowest birthweights. This gradation between the different smoking patterns was also evident for the head circumference and crown–heel length. Differences in length of gestation between the smoking categories were not found. Due to the low number of preterm births among smokers (N = 27), prematurity was not explored further. Similarly, there were too few LBW infants among smokers (N = 24) to conduct a meaningful analysis of this outcome.

The findings from the multivariable linear regression analyses can be seen in Table 4. These were restricted to the 697 women who had smoked at the time of their last menstrual period. As noted in the methods, the minimal sufficient adjustment set used was area deprivation, maternal age and maternal education for the outcomes explored. Similar to the pattern observed in the univariate analysis, a clear gradation in the anthropometric measurements between sustained quitters, partial quitters and sustained smokers was observed with sustained quitters having the most advantageous outcomes and sustained smokers the least. The gains for partial quitting were noteworthy: having adjusted for area deprivation, maternal age and maternal education, and compared with sustained smokers, babies born to partial quitters had birthweights that were 0.48 standard deviations (SD) (WHO z-score) heavier, head circumferences that were larger by 0.38 SDs (WHO z-score) and crown–heel lengths that were 0.23 SDs (WHO z-score) greater. Whereas sustained quitting yielded a small gain of 0.5 weeks on gestation, little gain was evident for partial quitting.

## 4. Discussion

### 4.1. Study Findings

This study addressed two hypotheses in relation to (1) the effect of sustained quitting and (2) the effect of partial quitting. It provides further evidence of the effectiveness of sustained quitting (hypothesis 1) on all outcomes examined. It also showed that partial quitting (hypothesis 2) showed some benefit over sustained smoking, but this was not as marked as for sustained quitting.

The work was conducted in a large, well-established cohort of pregnant women. Having adjusted for relevant sociodemographic and lifestyle factors using DAGs, a clear gradation in smoking patterns and anthropometric measures of foetal growth was demonstrated in line with our stated hypotheses. Women who were sustained quitters gave birth to infants with heavier birthweights, longer gestational age, larger head circumference and longer crown–heel length compared with sustained smokers who demonstrated the lowest values for these variables. For partial quitters, the values for these variables lay between those of sustained quitters and sustained smokers. Sustained quitting resulted in longer gestations than sustained smoking, but there was a minimal effect on gestation through partial quitting.

Our findings of increased birthweight in those who partially quit contribute to and support the growing research in this area, as outlined previously [14,18,19,20,21,22,23,24,25,26,27,28,29]. Even relatively modest gains in birthweight are important when addressing smoking in pregnancy from a population health perspective. The considerable gains in head circumference and crown–heel length support the robustness of our findings and are an important contribution to the relative dearth of studies of these measures to date [23,24,25,26,27]. Nonetheless, it should be noted that in the aforementioned studies, sustained quitting almost always resulted in better outcomes than partial quitting, as in our study, and this should remain the recommendation for all pregnant women who smoke.

The lack of any influence of partial quitting on gestation is unsurprising given the small number of preterm births (<37 weeks gestation) in the SWS cohort, along with the relatively small effect size observed to date [26,29]. It should be noted that a recent large meta-analysis of individual participant data from 28 pregnancy/birth cohorts in Europe and North America (including SWS) has shown that, compared with mothers who do not smoke, maternal first trimester smoking was not associated with adverse birth outcomes (SGA and preterm birth), although they were impacted upon by second and third trimester smoking [31].

The likely reason for the gradation in the effect of different smoking patterns in anthropometric birth outcomes lies in the understanding of the underlying mechanism by which cigarette smoke affects the placenta. At a physiological level, nicotine and its stable metabolite cotinine readily cross the human placenta and, thus, have direct toxic effects on the placenta, while other changes are likely due to the secondary vascular effects of tobacco smoke constituents, e.g., decreased flow of uterine blood to the placenta [4]. At a microscopic level, heavy smoking (e.g., 20 cigarettes per day) before 10 weeks of gestation had the greatest effects on placental morphology, suggesting that this is a particularly sensitive period [4]. Partial quitting likely functions by stopping this exposure for a period of time, albeit not to the same extent as sustained quitting.

### 4.2. Partial Quitting and other Harm Reduction Strategies in Practice

Our findings have particular relevance for socioeconomically disadvantaged women who smoke in pregnancy but find themselves unable to quit because of their circumstances. Within our cohort of women who were smokers at the time of pregnancy, there was a clear social gradient with those who continued to smoke during the second and third trimester, the sustained smokers, being most disadvantaged, the sustained quitters, most of whom will have stopped spontaneously on finding they were pregnant, the least disadvantaged, and partial quitters between the two, in keeping with other studies [7,8,9,14]. Our findings show that quitting for at least part of pregnancy would appear to be an effective strategy to improve birth outcomes, and this may be a message that continuing pregnant smokers may accept more readily. We note that learning one is pregnant serves as an important motivational cue for quitting attempts early in pregnancy and thus provides an impetus and an important window for health behaviour change, as many women are open to smoking cessation intervention at this time [51].

Notwithstanding our results, other harm reduction strategies have been proposed and warrant consideration. Smoking reduction, whereby women do not quit but reduce the number of cigarettes, and similarly tries to reduce chemical exposure. While some studies suggest a linear relationship between the number of cigarettes smoked and birthweight [52,53], most other studies, including more recent large cohort studies and meta-analyses, suggest that above 8–10 cigarettes, there is little effect suggesting even a few cigarettes make a profound difference [20,31,54]. Hence, reducing the number of cigarettes without quitting likely has limited beneficial effects on offspring and still exposes the mother to cigarette smoke.

In more recent times, electronic cigarettes (e-cigarettes) have been proposed as a harm reduction strategy, and while initial observational data is supportive of almost near-normal non-smoking birth outcomes [55], our understanding of the long-term effects of e-cigarettes use on both women and offspring, especially where chemical flavourings are used, is still largely unknown [56].

It should be noted, however, that it is unlikely that a single harm reduction strategy will be accepted by all women. Different harm reduction strategies will be needed based on the personal experience and circumstances of each woman.

### 4.3. Study Strengths and Limitations

This study has some notable strengths and limitations. It was conducted in a large established cohort of pregnant women with high completion rates for sociodemographic and smoking data. The use of DAGs allowed for careful consideration of confounders and appropriate adjustments using this available data. However, the main analysis focused on the smaller number of 697 women who smoked at the time of the last menstrual period, thus limiting the number available for the main analysis. This limited our ability to explore relationships between smoking patterns and low birth weight and preterm birth robustly. Furthermore, in studies that have shown a positive effect of partial quitting on gestation, the effect sizes have been small. Hence, this limiting of numbers may have also left the study insufficiently powered to detect an effect on gestation.

Although data on smoking status were collected during pregnancy and appropriate abstinence definitions were used, they were self-reported and not verified by biochemical measurement. Furthermore, the absence of second trimester smoking data meant it was not possible to examine trimester-specific quitting effects.

It should be noted that our baseline group of sustained smokers smoked more heavily before pregnancy than those who quit partially or fully. Therefore, it is possible that our findings are partly explained by the higher exposure to cigarette smoke in the early stages of pregnancy among sustained smokers. Although our adjustment included two socioeconomic variables, smoking is strongly socially patterned, and it is possible that residual confounding remains.

While some reported paternal variables were available, completion rates were low, and hence, they were not used in our study. Paternal variables may have had important confounding effects, especially on the anthropometric measurements. The recent aforementioned meta-analysis also examined the role of paternal smoking during pregnancy on birth outcomes and concluded that the evidence is currently unclear, but only SGA and preterm birth outcomes were examined [31].

Finally, it should be noted that the study is an observational one, so any attempts to infer causality must be made cautiously.

## 5. Conclusions

A significant minority of women continue to smoke during pregnancy [11,12]. Those who continue to smoke are often socioeconomically disadvantaged and tend to smoke more heavily (as confirmed in this study), and interventions aimed at sustained smokers need to recognise this [13]. Public health interventions should continue to focus on complete cessation, as this has been found consistently to have the most benefit on birth outcomes. Ideally, quitting would occur pre-conceptionally [57] especially given the lasting effects of maternal smoking on offspring growth [58] and epigenetic profile into adolescence [59].

Harm reduction strategies, however, may represent a way of reducing some of the adverse effects of smoking on offspring by working within the confines and difficulties experienced by these women both in terms of their social circumstances and their degree of smoking dependence. Any cessation of smoking, especially at the start of pregnancy or during pregnancy, and ideally before pregnancy, has the potential to have positive birth outcomes. This study provides further support for partial quitting as a practical harm reduction strategy for women who are currently unable to cease smoking for the duration of their pregnancy, the majority of whom are in lower socioeconomic groups and suffer multiple environmental stresses related to their socioeconomic position.

## Figures and Tables

**Table 1:** Maternal demographic characteristics according to maternal smoking status (total N = 3158). Results expressed as mean (standard deviation), median (interquartile range), number (percentage (%)) as appropriate.

	Non-Smokers (N = 2461)	Smokers (N = 697)	Sustained Smokers (N = 355)	Partial Quitters (N = 81)	Sustained Quitters (N = 261)
Age (years) (mean, SD)	31.0 (3.7)	29.4 (4.2)	29.5 (4.1)	29.4 (4.7)	29.2 (4.1)
Ethnicity (N, %)					
White	2332 (94.8)	684 (98.1)	349 (98.3)	78 (96.3)	257 (98.5)
Non-White	128 (5.2)	13 (1.9)	6 (1.7)	3 (3.7)	4 (1.5)
Gestational Diabetes Mellitus (N, %)	36 (1.5)	2 (0.3)	1 (0.3)	1 (1.2)	0 (0.0)
Maternal Pre-pregnancy Body Mass Index (kilograms/metres^2^) (median, IQR)	24.1 (21.9, 27.3)	24.3 (21.9, 27.7)	24.5 (22.0, 28.0)	25.3 (22.5, 28.9)	23.8 (21.5, 26.7)
Primiparous (N, %)	1093 (51.5)	295 (42.3)	114 (32.1)	36 (44.4)	145 (55.6)
Townsend Deprivation Index Score (mean, SD)	−0.2 (3.1)	1.0 (3.3)	1.4 (3.2)	1.1 (3.5)	0.5 (3.2)
Receiving Benefits (N, %)	263 (10.7)	215 (30.8)	153 (43.1)	15 (18.5)	47 (18.0)
Educational Attainment (N, %)					
None	49 (2.0)	50 (7.2)	32 (9.0)	5 (6.3)	13 (5.0)
Certificate of Secondary Education	182 (7.4)	113 (16.2)	75 (21.1)	13 (16.3)	25 (9.6)
O-levels	651 (26.5)	260 (37.4)	146 (41.1)	22 (27.5)	92 (35.2)
A-levels	762 (31.1)	191 (27.4)	86 (24.2)	30 (37.5)	75 (28.7)
Higher National Diploma	172 (7.0)	26 (3.7)	6 (1.7)	4 (5.0)	16 (6.1)
Degree or above	637 (26.0)	56 (8.0)	10 (2.8)	6 (7.5)	40 (15.3)
Early-pregnancy Prudent Diet Score (z-score) (mean, SD)	0.2 (0.9)	−0.6 (1.0)	−0.9 (0.9)	−0.4 (1.1)	−0.2 (1.0)

Abbreviations: IQR interquartile range; SD standard deviation.

**Table 2:** Maternal smoking characteristics according to maternal smoking status.

	All Smokers (N = 697)	Sustained Smokers (N = 355)	Partial Quitters (N = 81)		Sustained Quitters (N = 261)
			First Trimester Quitters (N = 32)	Third Trimester Quitters (N = 49)	
Age commenced smoking (years) (median, IQR)	16 (14–17)	15 (14–17)	16 (15–17)	16 (14–18)	16 (15–18)
Cigarettes/day at the last menstrual period (median, IQR)	15 (7–20)	18 (10–20)	11 (8–18)	10 (6–20)	7 (4–15)
Cigarettes/day in the first trimester (median, IQR)	10 (5–10)	10 (5–12)	-	5 (2–6)	-
Cigarettes/day in the third trimester (median, IQR)	10 (5–15)	10 (5–15)	3 (2–10)	-	-

Results expressed as median (interquartile range). Abbreviation: IQR interquartile range.

**Table 3:** Infant characteristics according to maternal smoking status. Results expressed as mean (standard deviation), median (interquartile range) as appropriate.

	Non-Smokers (N = 2461)	Smokers (N = 697)	Sustained Smokers (N = 355)	Partial Quitters (N = 81)	Sustained Quitters (N = 261)
Birthweight (WHO z-score) (mean, SD)	0.1 (1.0)	−0.1 (1.0)	−0.4 (1.0)	0.1 (1.0)	0.2 (0.9)
Head circumference (WHO z-score) (mean, SD)	0.4 (1.0)	0.3 (1.0)	0.1 (1.0)	0.5 (1.1)	0.5 (1.1)
Crown–heel length (WHO z-score) (mean, SD)	−0.3 (0.8)	−0.5 (0.9)	−0.7 (0.9)	−0.5 (1.0)	−0.2 (0.8)
Gestation (weeks) (median, IQR)	40.0 (39.0, 41.0)	40.1 (39.1, 41.0)	39.8 (38.9, 40.7)	40.0 (38.9, 41.0)	40.3 (39.6, 41.1)

Abbreviations: IQR interquartile range; SD standard deviation; WHO World Health Organisation.

**Table 4:** Infant characteristics (adjusted *) according to maternal smoking status compared with sustained smokers as baseline.

		Partial Quitters		Sustained Quitters	
	N	β	95% CI	β	95% CI
Birthweight (WHO z-score)	686	0.48	(0.24–0.72)	0.64	(0.47–0.80)
Head circumference (WHO z-score)	679	0.38	(0.12–0.64)	0.41	(0.23–0.59)
Crown–heel length (WHO z-score)	671	0.23	(0.02–0.45)	0.54	(0.40–0.45)
Gestation (weeks)	691	0.10	(−0.25–0.45)	0.50	(0.26–0.74)

* Adjusted for area deprivation, maternal age and maternal education with baseline of sustained smokers. Abbreviations: β regression coefficient; CI confidence interval; WHO World Health Organisation.

## Data Availability

Due to ethical concerns and original agreements made with participants, supporting data cannot be made openly available. The SWS team can provide the data on request subject to appropriate approvals. Researchers wishing to use the data would need to make a formal application to the SWS Oversight Group through the cohort PI and co-author of this paper—Hazel Inskip: hmi@mrc.soton.ac.uk and ensure appropriate ethical approval is in place. Subject to approval and formal agreements being signed, the data would then be provided.

## Supplementary Materials

The following are available online at https://www.mdpi.com/1660-4601/17/21/7991/s1, Figure S1. Directed Acyclic Graph (DAG) (with legend) showing the relationship between smoking in pregnancy and birthweight (z-score), along with possible confounders; Figure S2. Directed Acyclic Graph (DAG) (with legend) showing the relationship between smoking in pregnancy and head circumference (z-score), along with possible confounders; Figure S3. Directed Acyclic Graph (DAG) (with legend) showing the relationship between smoking in pregnancy and crown–heel length (z-score), along with possible confounders; Figure S4. Directed Acyclic Graph (DAG) (with legend) showing the relationship between smoking in pregnancy and gestation, along with possible confounders.
