# Supplementary material for: The Effects of Different Smoking Patterns in Pregnancy on Perinatal Outcomes in the Southampton Women’s Survey
Source: Int J Environ Res Public Health. 2020 Oct 30;17(21):7991. doi: 10.3390/ijerph17217991 (PMC7663677; doi:10.3390/ijerph17217991)
Supplement: Supplementary file 1 [file ijerph-17-07991-s001.pdf]

International Journal of
Environmental Research and Public Health

MDPI

Supplementary Materials

The Effects of Different Smoking Patterns in Pregnancy on Perinatal Outcomes in the Southampton Women's Survey

Martin M. O'Donnell, Janis Baird, Cyrus Cooper, Sarah R. Crozier, Keith M. Godfrey, Michael Geary, Hazel M. Inskip and Catherine B. Hayes

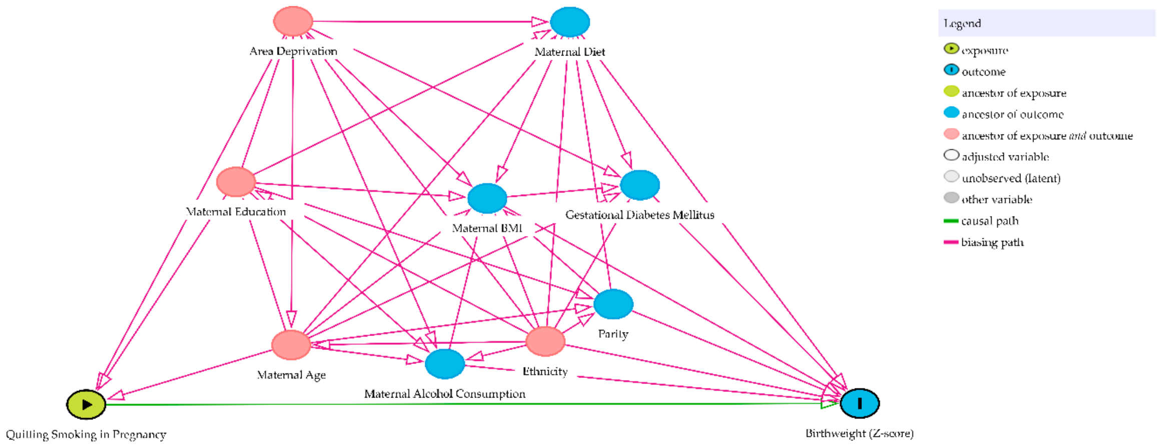

Figure S1. Directed Acyclic Graph (DAG) (with legend) showing the relationship between smoking in pregnancy and birthweight (z-score), along with possible confounders. Abbreviation: BMI body mass index.

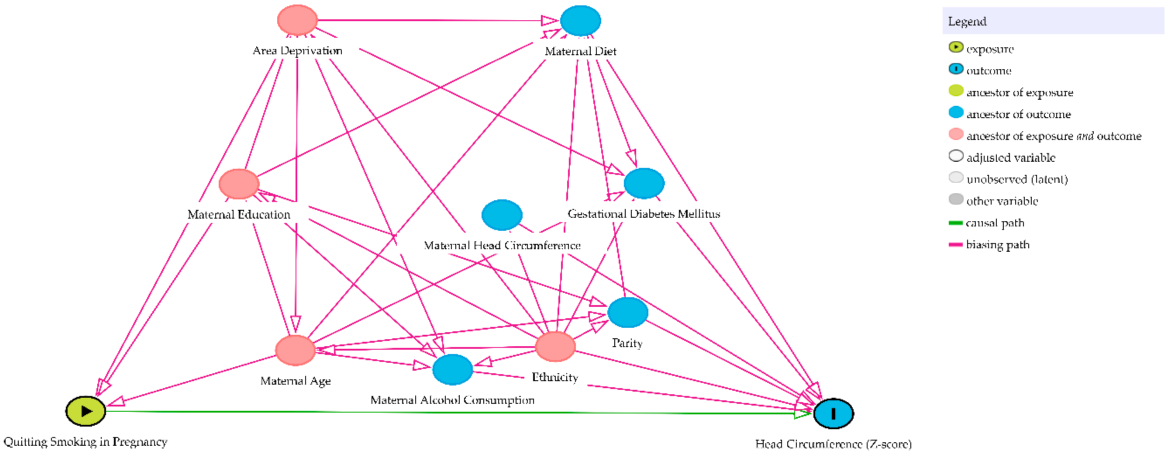

Figure S2. Directed Acyclic Graph (DAG) (with legend) showing the relationship between smoking in pregnancy and head circumference (z-score), along with possible confounders.

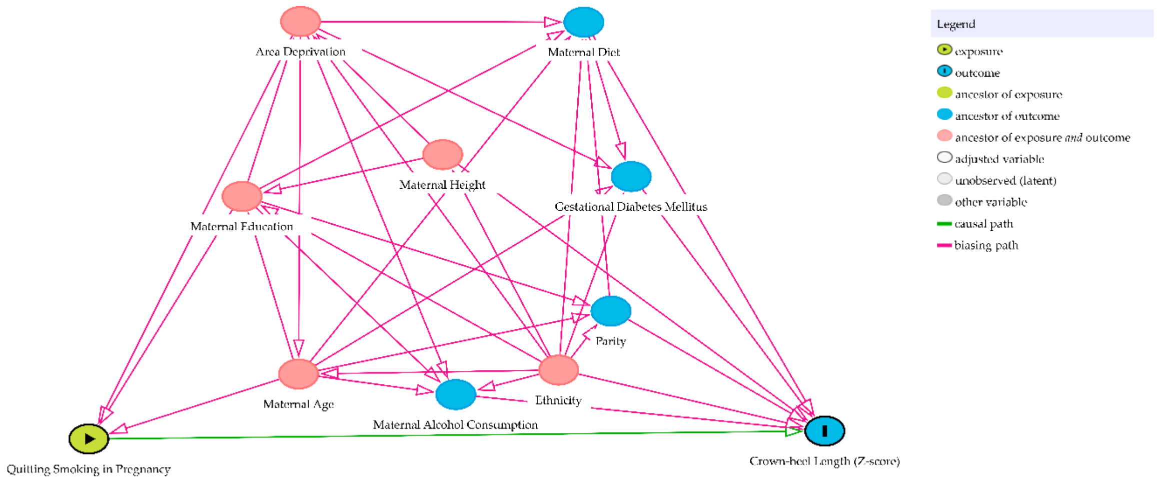

Figure S3. Directed Acyclic Graph (DAG) (with legend) showing the relationship between smoking in pregnancy and crown-heel length (z-score), along with possible confounders.

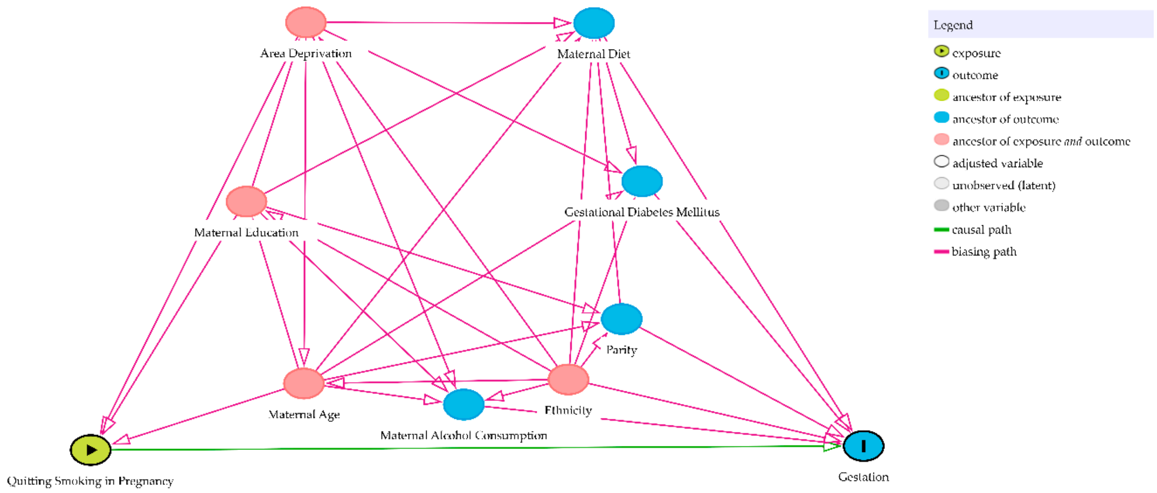

Figure S4. Directed Acyclic Graph (DAG) (with legend) showing the relationship between smoking in pregnancy and gestation, along with possible confounders.

© 2020 by the authors. Submitted for possible open access publication under the terms and conditions of the Creative Commons Attribution (CC BY) license (http://creativecommons.org/licenses/by/4.0/).
